# Editorial: Parental questionnaires as a reliable instrument for the assessment of child language development

**DOI:** 10.3389/fpsyg.2024.1471747

**Published:** 2024-09-02

**Authors:** Maria-José Ezeizabarrena, Melita Kovacevic

**Affiliations:** ^1^Departement of Linguistics and Basque Studies, University of the Basque Country (UPV/EHU), Vitoria-Gasteiz, Spain; ^2^ELEBILAB, Bilingualism Research Laboratory (UPV/EHU), Vitoria-Gasteiz, Spain; ^3^Laboratory for Psycholinguistic Research, Department for Speech and Language Pathology, ERF, University of Zagreb, Zagreb, Croatia

**Keywords:** parental questionnaire, child language, development, assessment, vocabulary, grammar, pragmatics

Parents and caregivers have the experience of observing their children many hours per week, including school days, weekends and holidays, and in multiple situations of tiredness and lack of attention, but also of excitement, happiness and creativity. Such a varied experience makes them the ideal informants about children's behavior. Therefore, professionals such as pediatricians, psychologists, speech and language therapists, and teachers take great note of the information provided by the parents when assessing their children's health, general and learning skills and communicative abilities.

One of the tools used widely in the assessment of language development and in psycholinguistic research to compile such valuable information is questionnaires. Except for the time needed to fill them out, parental questionnaires do not require a major effort. Informants do not need to disrupt their daily life by scheduling appointments with the professional either, since they can select the time and place in which they feel more comfortable reporting on their children's linguistic experience and verbal (and non-verbal) communicative skills. Moreover, data obtained using this methodology are less likely to be influenced by factors that may mask a child's “true” abilities in the laboratory or clinic, such as child's non-compliance, shyness or time limitations.

This volume presents studies conducted with different types of parental questionnaires in either their original version or in their adaptations to other languages. It comprises fifteen articles—12 original research, one brief research, one mini-review and one opinion—on young children's language development. Twelve of them are based on *The MacArthur-Bates Communicative Development Inventories* (CDI) (Fenson et al., 2007). The remaining three used, respectively, the *Language Use Inventory* (LIU) by O'Neill (2009), the *Parents of Bilingual Children Questionnaire* (PaBiQ) by Tuller (2015), or the *Parental Linguistic Concern Questionnaire* (PLCQ), based on Restrepo (1998).

Five CDI questionnaires are available in many languages for the assessment of infants' and toddlers' communicative skills at different ages. All these five instruments were originally developped to norm the non-verbal and/or verbal communicative skills in English of (mostly monolingual) infants and toddlers living in the USA. The long *Words and Gestures* (CDI-1) was designed to test gestures, receptive and expressive vocabulary of 8–15 months (originally), whilst the age range has been extended to 18 month-olds or even older in some of the adaptations. Its shorter version, CDI-1sh, tests vocabulary only. The *Words and Phrases* (CDI-2) questionnaire was designed to test expressive vocabulary and grammar of 16- to 24 month-olds, although its short version (CDI-2sh) only tests vocabulary. Finally, the CDI-3, of which there is only a short version, was originally designed to test vocabulary, grammar and language use of children up to age 4 (Fenson et al., 2007). In their opinion article, Marchman and Dale present a comprehensive overview of the contribution of the samplings conducted in the late 20^th^ century with the original USA-English CDI-2 printed versions, and compare those findings with more recent ones, obtained in the current century from new child populations and using online procedures. Despite slight differences demonstrated, rates of vocabulary size and increase between age 16 and 30 months appear as very consistent across samples, confirming the robustness of the data and the reliability of the instrument.

CDI instruments have been adapted to over 100 languages in the world. The number of 12 (mostly European) countries and language varieties involved in this Research Topic is a clear evidence of the international impact of CDIs and their adaptations. Data from Finno-Ugric languages such as Estonian (Tulviste and Schults) and Finnish (Surakka et al.) are presented in addition to Germanic languages, such as British English (Jago et al.), Norwegian (Holm et al.), and Swedish (Eriksson and Myrberg), to Romance languages, such as Catalan (Feijoo et al.), Chilean Spanish (Varela-Moraga et al.), and Galician (Ogneva and Pérez-Pereira), to Semitic languages, such as Hebrew (Ohana and Armon-Lotem) and Maltese (Gatt et al.) and to Slavic languages, such as Croatian (Šmit Brlekovič and Kuvač-Kraljevič).

Some of the CDI articles deal with typically developing, and almost exclusively monolingual, children (Holm et al.; Jago et al.; Šmit Brlekovič and Kuvač-Kraljevič; Marchman and Dale; Surakka et al.; Tulviste and Schults; Varela-Moraga et al.). Others report on children with or at risk of developmental delay (Eriksson and Myrberg; Jago et al.; Šmit Brlekovič and Kuvač-Kraljevič; Ogneva and Pérez-Pereira; Varela-Moraga et al.). A set of papers report on and explore the ways of (better) assessing the linguistic development of children with bi- or multilingual language exposure and use (Eriksson and Myrberg; Ohana and Armon-Lotem) or compare the acquisition of the same language in normal and exceptional pandemic circumstances (Feijoo et al.). Variation was found across CDI studies in participants' profiles, but also in the specific questionnaire used in their assessment. Some used the CDI-1 (Feijoo et al.; Jago et al.; Surakka et al.; Varela-Moraga et al.), alone or together with the CDI-2 (Feijoo et al.; Gatt et al.; Marchman and Dale; Ogneva and Pérez-Pereira; Ohana and Armon-Lotem; Surakka et al.; Varela-Moraga et al.), whilst others used the CDI-3 (Eriksson and Myrberg; Holm et al.; Šmit Brlekovič and Kuvač-Kraljevič; Tulviste and Schults). The majority of papers converge in testing and demonstrating the internal consistency and validity of the instruments across languages. Some provide additional evidence of their validity to predict outcomes even over 2 years later.

Vocabulary and grammar are not the only linguistic components assessed through parental questionnaires. The mini-review by Pesco and O'Neill presents the *Language Use Inventory* (LIU), an instrument designed to measure children's pragmatic knowledge, originally in English, and an overview of its adaptation to seven additional languages, namely Arabic, French, Italian, Mandarin, Norwegian, Polish, and Portuguese. Based on the instrument's sensitivity to age and its usefulness across different linguistic and cultural contexts, Pesco and O'Neil conclude that LIU is valuable for clinical and research purposes.

Auza et al.'s paper analyses the strengths and weaknesses of the *Parental Language Concern Questionnaire* (PLCQ) in the identification of monolingual Mexican Spanish-speaking children with delay in language development. They conclude that a reduced questionnaire conformed by four out of the eight items in the list, in combination with one of the four items of the additional list of *Biological and Environmental Conditions Questions*, based on Peñaloza (2018) is a reliable screening method for identifying children with language disorders.

The usefulness of parental questionnaires extends to the assessment of older than pre-school aged children, as demonstrated by Pourquié et al. in their investigation, in which data obtained with the parental questionnaire HEGA (Haur Elebidunen Gurasoentzako Galdetegia ‘Questionnaire for parents of bilingual children'), the Basque adaptation of *Parents of Bilingual Children Questionnaire* (PABIQ) were tested against performance data in Basque of 4- to 9-year-old children. They found a correlation between the parental responses to questions on their children's linguistic experience and children's accuracy at several scales of expressive vocabulary and grammar in Basque.

The studies compiled in this volume confirm: (a) the interest of the community of researchers and professionals of language therapy for the development and use of parental questionnaires to assess language development; (b) the consistency of the data, inter-individually, intra-individually and across languages; and (c) the reliability, validity, and usefulness of these tools for identifying atypical development in children's early and later communicative skills.

## Author contributions

M-JE: Writing – original draft, Writing – review & editing. MK: Writing – review & editing.
